# Mechanisms and Efficacy of Transcranial Stimulation Technologies in Substance Use Disorders: A Focus on Prefrontal Cortex Stimulation

**DOI:** 10.1192/j.eurpsy.2025.2128

**Published:** 2025-08-26

**Authors:** C. Pinheiro Ramos, A. F. Reis, M. J. Freire, S. Mendes

**Affiliations:** 1 Setúbal Hospital Center, Setúbal, Portugal

## Abstract

**Introduction:**

About 31 million people worldwide suffer from substance use disorders (SUDs), causing significant health and economic burdens.

SUDs are linked to reduced dopamine activity in the mesolimbic region of the brain, as well as dysfunction in the dorsolateral prefrontal cortex (DLPFC) and dorsal anterior cingulate cortex (dACC), which are responsible for decision-making and self-control. Additionally, the ventral prefrontal cortex (PFC), including the orbitofrontal cortex (OFC) and ventral anterior cingulate cortex (vACC), play a role in emotional processing and limbic arousal.

A promising approach to treating SUDs involves non-invasive neuromodulation techniques (NIBS), specifically repetitive transcranial magnetic stimulation (rTMS) and transcranial direct current stimulation (tDCS).

**Objectives:**

To better understand the role of non-invasive neuromodulation techniques in substance use disorders.

**Methods:**

A search was conducted in various databases, including PubMed.

**Results:**

Many studies using rTMS to treat SUDs have targeted the DLPFC. When the left DLPFC is stimulated, the effects are generally positive, and the treatment produces clinically significant results for tobacco, stimulant, and opioid use disorders.

It has been found that the medial PFC (mPFC) could be a potential target for therapy, especially when using deep TMS, as demonstrated by studies involving alcohol and cocaine. Both the DLPFC and mPFC are promising targets for rTMS.

Regarding tDCS, it seems that right anodal DLPFC stimulation is the most effective method across all types of substances.

**Conclusions:**

Much remains unknown regarding the mechanisms by which rTMS or tDCS induce therapeutic effects in SUDs. Further research is necessary to determine the clinical safety and efficacy of these treatments.

**Disclosure of Interest:**

None Declared

